# Electrically tunable artificial gauge potential for polaritons

**DOI:** 10.1038/ncomms14540

**Published:** 2017-02-23

**Authors:** Hyang-Tag Lim, Emre Togan, Martin Kroner, Javier Miguel-Sanchez, Atac Imamoğlu

**Affiliations:** 1 Institute of Quantum Electronics, ETH Zurich, CH-8093 Zurich, Switzerland

## Abstract

Neutral particles subject to artificial gauge potentials can behave as charged particles in magnetic fields. This fascinating premise has led to demonstrations of one-way waveguides, topologically protected edge states and Landau levels for photons. In ultracold neutral atoms, effective gauge fields have allowed the emulation of matter under strong magnetic fields leading to realization of Harper-Hofstadter and Haldane models. Here we show that application of perpendicular electric and magnetic fields effects a tunable artificial gauge potential for two-dimensional microcavity exciton polaritons. For verification, we perform interferometric measurements of the associated phase accumulated during coherent polariton transport. Since the gauge potential originates from the magnetoelectric Stark effect, it can be realized for photons strongly coupled to excitations in any polarizable medium. Together with strong polariton–polariton interactions and engineered polariton lattices, artificial gauge fields could play a key role in investigation of non-equilibrium dynamics of strongly correlated photons.

## Abstract (web summary)

Artificial gauge fields promise a route to controlling topological properties of photonic systems but have only been realized by static design. Here, Lim *et al*. demonstrate that perpendicular electric and magnetic fields can effect dynamically controlled artificial gauge potentials for polaritons.

Synthesis of artificial gauge fields for photons have been demonstrated in a number of different optical systems. In most cases, the implementation is achieved through the design of the optical system^1^,^2^,^3^,^4^, leaving little or no room for fast control of the magnitude of the effected gauge field after sample fabrication is completed. For many applications on the other hand, it is essential to be able to tune or adjust the strength of the gauge field during the experiment^5^,^6^,^7^; this is particularly the case for nanophotonic structures^8^ where fast local control of the gauge field strength may open up new possibilities for investigation of many-body physics of light^9^.

Cavity-polaritons are hybrid light-matter quasi-particles arising from non-perturbative coupling between quantum well (QW) excitons and cavity photons. A magnetic field 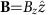 applied along the growth direction influences polaritons through their excitonic nature and leads to a diamagnetic shift, Zeeman splitting of circularly polarized modes and enhancement of exciton–photon coupling strength^10^. For excitons with a non-zero momentum, the applied *B*_*z*_ also induces an electric dipole moment^11^,^12^,^13^,^14^. In a classical picture, this dipole moment is due to the Lorentz force that creates an effective electric field **E**_eff_ (ref. 15) that is proportional to **k** × **B**. For excitons with small momentum and polarizability *α*, this **E**_eff_ causes an induced dipole moment (**d**∝*α***k** × **B**). An additional external electric field **E**_ext_ in the QW plane then alters the dispersion of excitons as it leads to energy changes proportional to *k*:−**d**·**E**_ext_∝−*α*(**k** × **B**)·**E**_ext_, so that the energy minimum is no longer at |**k**|=0 as would be the case for a free particle with an effective mass but at finite **k**. This simple modification of the dispersion relation is equivalent to an effective gauge potential **A**_eff_ for excitons; due to their partly excitonic character, the Hamiltonian describing the dynamics of polaritons also contains an effective gauge potential term proportional to **A**_eff_.

In the realization we describe here, based on the effective gauge potential **A**_eff_, the strength and direction of the effected gauge potential is controlled electrically. Moreover, since our scheme relies on the magnetoelectric Stark effect, time reversal symmetry^16^,^17^,^18^ of the optical excitations is broken. This realization, in combination with strong polariton–polariton interactions^19^,^20^,^21^, has the potential to open up new possibilities of investigating many-body physics of light.

## Results

### Demonstration of the effective electric field **E**_eff_

**Figure 1:**
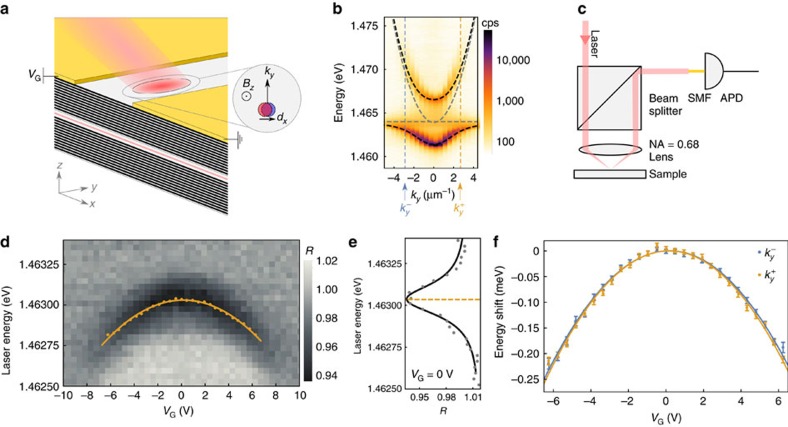
Description of the sample and characterization experiments at *B*_*z*_=0 T. (**a**) The sample, held at 4 K in a helium bath cryostat, contains three In_0.04_Ga_0.96_As quantum wells located at an antinode of a cavity formed by two DBRs. An electric potential *V*_G_ applied to the metal gates deposited on the sample surface creates an electric field in the *x* direction (see ‘Methods' section). Inset: with a magnetic field in the *z* direction polaritons excited with in-plane wavevector *k*_*y*_ exhibit a dipole moment *d*_*x*_. (**b**) *k*_*y*_-resolved PL spectra at *B*_*z*_=0 T. Grey dashed lines show the extracted bare cavity and exciton dispersions, black lines show polariton dispersions (see Supplementary Note 3 for parameters), orange and blue lines correspond to *k*_*y*_=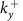 and *k*_*y*_=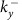, respectively. cps stands for counts per second. (**c**) Experimental set-up used in reflection measurements. Reflection of a linearly polarized laser beam (<100 pW) with in-plane wavevector *k*_*y*_ is coupled to a single-mode fibre (SMF) and its intensity is detected with an avalanche-photodiode (APD). (**d**) Changes in the reflected intensity, *R*, of a laser beam exciting polaritons with in-plane wavevector 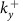 as a function of laser energy and *V*_G_. Yellow dots indicate the extracted polariton resonance energies. Note that the polariton signal is missing (|*V*_G_|>8 V) due to the ionization of the exciton. (**e**) Line cut of the reflection spectrum at *V*_G_=0 V. Grey points are the measured reflection data, black solid line shows the fitted lineshape (see Supplementary Note 3). Dashed yellow line indicates the extracted polariton resonance energy. (**f**) Change of polariton energy from *V*_G_=0 V, as a function of *V*_G_ for polaritons excited with 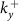 (yellow) and 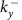 (blue) at *B*_*z*_=0 T, solid lines are fits to second-order polynomials (blue: 2.1 × 10^−6^ *V*_G_−5.6 × 10^−6^ 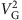, yellow: 1.9 × 10^−6^ *V*_G_−5.8 × 10^−6^ 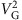). Error bars represent 1 s.d. of the polariton resonance energy extracted from a fit of the data to a reflection lineshape (see Supplementary Note 3).

The cavity polariton sample we use is illustrated in Fig. 1a. Our demonstration relies on the fact that it is possible to excite polaritons with a well-defined in-plane wavevector **k** by appropriately choosing the angle and energy of the excitation beam. As illustrated in Fig. 1a, we choose 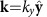. Two metal gates deposited 30 μm apart allow us to apply an electric field in the *x* direction such that for polaritons propagating with a wavevector along the *y* direction, 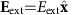 will add or cancel 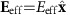 due to the Lorentz force. By recording changes in the transition energy of polaritons as a function of *E*_ext_, we can determine the strength of *E*_eff_. In our experiments, we only address the lower polariton branch.

Changes in the reflected intensity of a laser beam probing polaritons at *B*_*z*_=0 T with 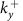
=2.7 μm^−1^ as a function of *E*_ext_ is shown in Fig. 1d. For each *E*_ext_, the reflected intensity shows a dip at an energy corresponding to the polariton resonance (Fig. 1e). The spectral centre of the dip shifts to lower energies with *E*_ext_ in a way that is well described by a second-order polynomial. The expected behaviour for a neutral polarizable quasi-particle (that is, exciton or polariton) that is subject to *E*_ext_, would be to have a dc-Stark shift equal to −*α*|*E*_ext_|^2^ (ref. 22); here the coefficient *α* is the quasi-particle polarizability. Therefore, the electric field that yields the maximum lower-polariton energy identifies 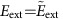 that exactly cancels any internal or effective electric fields *E*_eff_ 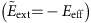. To quantify *E*_eff_ due to the Lorentz force described above, we extract the difference of 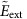 at a fixed *B*_*z*_ for polaritons excited with 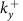
=2.7 μm^−1^ and 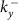
=−2.9 μm^−1^. This approach also allows us to exclude influences of built-in electric fields. At *B*_*z*_=0 T, we find that the difference of *V*_G_ values that correspond to the maximum energy with 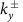 excitation is 0.02 V, indicating that *E*_eff_ is negligible at 0 T (Fig. 1f).

**Figure 2:**
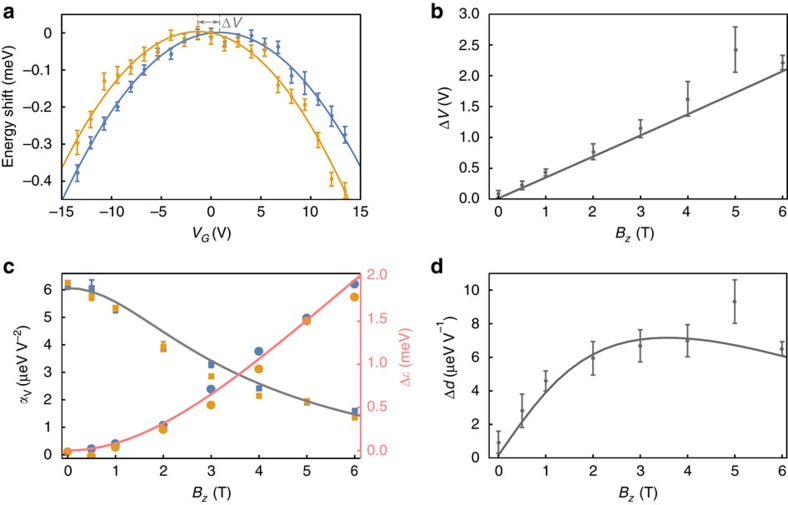
Magnetic field dependence. (**a**) Shift of polariton energies with applied voltage for polaritons excited with 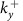 (yellow) and 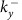 (blue) at *B*_*z*_=5 T; solid lines are fits to second-order polynomials (−*d*_*V*_*V*_G_−*α*_*V*_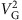, blue: *d*_*V*_=3.0 × 10^−6^ eV V^−1^, *α*_*V*_=1.8 × 10^−6^ eV V^−2^, yellow: *d*_*V*_=−5.2 × 10^−6^ eV V^−1^, *α*_*V*_=1.9 × 10^−6^ eV V^−2^). Error bars represent 1 s.d. of the polariton resonance energy extracted from a fit of the data to a reflection lineshape (see Supplementary Note 3). Grey dashed lines indicate the voltage at which maximum energy occurs for the two parabolas, the grey arrow indicates the difference between them (Δ*V*). (**b**) Using data such as **a** at different *B*_*z*_, difference between the extracted voltage (Δ*V*) at which maximum energy occurs for 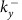 and 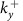 excitations. (**c**) Change in *α*_*V*_ (polarizability) with *B*_*z*_ (filled squares, left axis, and grey curve). Change of lower polariton resonant energy with *B*_*z*_ at *V*_G_=0 V (filled circles, right axis, and red curve) from the value at *B*_*z*_=0 T. Yellow and blue data points are data for 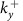 and 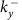, respectively. (**d**) Difference between extracted *d*_*V*_ values (electric dipole moment) for polaritons propagating with 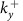 and 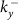. For **b**–**d**, error bars are the estimated standard deviations of the mean for three repetitions of the experiment (some error bars are smaller than the markers), and the solid lines are results of a numerical calculation (see Supplementary Note 4).

In stark contrast, for *B*_*z*_=5 T, the energy shift of polaritons with *E*_ext_ displays a significant difference between 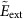 for 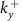 and 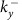, as illustrated in Fig. 2a. The magnetic field dependence of the difference of 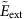 for 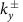, Fig. 2b, shows a behaviour that is well described by a linear increase with *B*_*z*_, demonstrating *E*_eff_ due to the Lorentz force.

### *B*_*z*_-induced changes to polaritons

With increasing *B*_*z*_, the polarizability decreases and the polariton energy at *E*_ext_=0 increases, as illustrated in Fig. 2c. The polariton energy at *E*_ext_=0 and the polarizability can be extracted from a fit to a second-order polynomial: 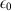
−*dE*_ext_−*α*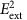. The first-order coefficient of the polynomial in turn, yields the induced dipole moment of the polaritons (excitons). The difference of the first-order coefficient for ±*k*_*y*_ excitation beams (twice the induced dipole moment) is shown in Fig. 2d: we observe that the induced dipole moment first increases with *B*_*z*_ and then starts to slowly decrease for *B*_*z*_≥4 T. We expect that for the high magnetic field regime, the induced dipole moment decreases as 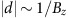 (ref. 12). We find very good agreement with a theoretical model of the polaritons (shown as solid lines in Fig. 2) that allows us to identify the physical mechanism underlying the overall *B*_*z*_ and *E*_ext_ dependence (see Supplementary Note 4 for detailed information about the model). The change in the lower polariton energy is due to an interplay between a diamagnetic blue-shift of the exciton transition energy and a red-shift due to an increase in the cavity-exciton coupling strength^10^. Concurrently, the polarizability decreases with *B*_*z*_, as would be expected in a classical picture as the size of the polarizable particle decreases. We emphasize that due to the change in the exciton energy and cavity-exciton coupling strength the exciton content of the polaritons changes as *B*_*z*_ is varied. We also find that the energy shift of polaritons with *E*_ext_ is influenced by the decrease in the electron–hole overlap due to *E*_ext_-induced polarization, which leads to a reduction of the cavity-exciton coupling strength. In the *E*_ext_ range that we probe the decrease in the cavity-exciton coupling leads to an effective smaller polarizability for polaritons as compared to excitons, due to a reduction on the excitonic character of the polaritons. Moreover, the model also captures the presence of *E*_eff_ due to the centre of mass motion of polaritons and confirms that even with changes in exciton content with *B*_*z*_, its expected dependence on *B*_*z*_ is still linear. Finally, we emphasize that while *E*_eff_ arises from exciton physics, the strong coupling between excitons and photons has to be taken into account to obtain a quantitative agreement between the model and our experiments.

### Demonstration of a gauge potential for polaritons

Remarkably, our findings demonstrate that polariton energy under perpendicular **B** and **E**_ext_ depends not only on the magnitude of **k** but also on its direction: this non-reciprocal flow of light is an indication of the presence of a gauge field for polaritons. The dispersion relation for free excitons propagating in the QW plane with wavevector 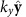 in the presence of an electric field 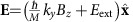 is:


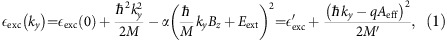


where *M* is the total exciton mass, and 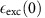 is the exciton energy at *k*_*y*_=0 and *E*_ext_=0. 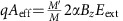 is an effective vector potential for excitons, with 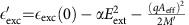 and 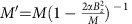. The strong coupling to the cavity further changes the dispersion relation, but polaritons in a narrow energy-momentum range can still be treated as free particles under the influence of an effective vector potential for polaritons, *qA*, whose value depends on the detuning between the excitons and polaritons as well as their effective masses (see Supplementary Note 4).

For our experiments, both *B*_*z*_ and *E*_ext_ are uniform over the area of our experiment yielding a constant gauge potential *qA*. Since physical observables are gauge invariant, we might expect that a constant gauge potential would not have any observable experimental signatures, and our measurements to be independent of *qA*. In practice, our experiments involve propagation of photons between two regions with different constant gauge potentials; when calculating the magnitude of *qA* above, we fixed a gauge by choosing the constant gauge potential for photons outside the cavity to be *qA*_out_=0. The difference *qA*−*qA*_out_≠0 is gauge invariant and stems from an effective (sheet) magnetic field at the interface between the cavity and vacuum. The experimental signatures of *qA*, such as shift of the energy minimum of polariton dispersion away from *k*=0, are observable in our experiments due to this effective magnetic field that alters the kinetic momentum as photons/polaritons pass through the interface^23^,^24^,^25^.

**Figure 3:**
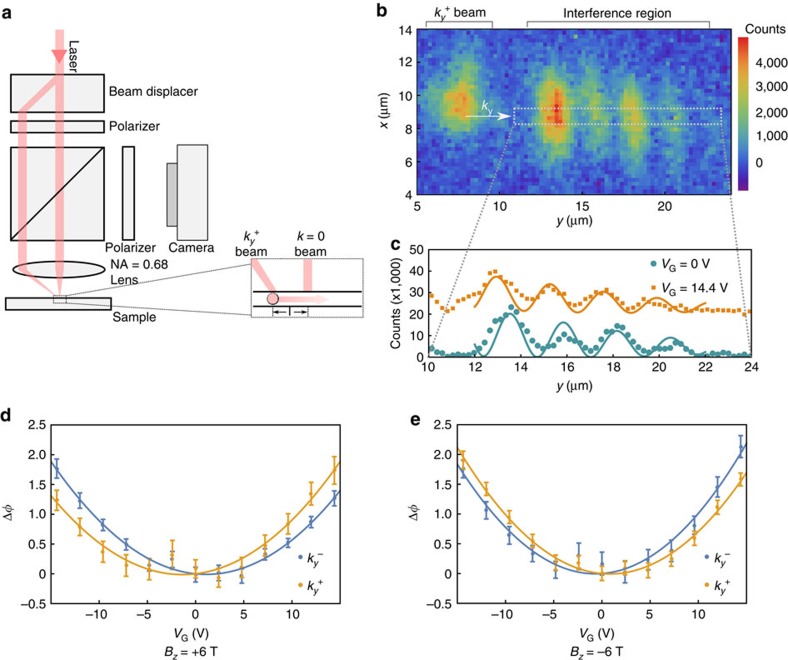
Demonstration of effective gauge potential for propagating polaritons. (**a**) A single laser beam is split into two using a birefringent beam displacer, ensuring the relative phase between the two beams does not fluctuate during the experiment. Resulting two linearly polarized beams (total power 40 nW) are incident at different positions on the high numerical aperture (NA=0.68) lens. One beam that is 1.2 mm off from the centre of the lens is incident on the sample with 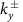. The second beam passes through the centre of the lens and thus has vanishing in-plane momentum (*k*=0 beam). The sample-lens distance is less than the focal length of the lens to ensure that the two beams are incident on the sample with their centres displaced by *l* ∼9 μm. To detect polariton flow, we use an additional linear polarizer between the camera and sample that transmits light that is nearly orthogonally polarized to the incident beams. (**b**) An interference image obtained at *B*_*z*_=−6 T, *V*_G_=0 V. Polaritons excited by the 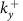 beam at 1.46467, eV propagate in the direction indicated by the white arrow. In the spatial region where photons emitted by propagating polaritons overlap with the reflected *k*=0 beam an interference pattern is observed. (**c**) In green, sum of the detected intensity of the interference pattern shown in **b** in the range *x*=[8.5−9.4] μm. In yellow, same as for green but shifted in intensity, for an image obtained at *V*_G_=14.4 V. Solid lines are fits to the model described in ‘Methods' section and show a difference in the extracted phase. (**d**) Change in the extracted phase at *B*_*z*_=6 T as a function of *V*_G_ for polaritons excited with 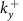 (yellow) and 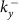 (blue). Solid lines show second-order polynomial fits to the phase change as a function of *V*_G_ (blue: −0.016 *V*_G_+0.0072 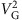, yellow: 0.019 *V*_G_ + 0.0071 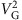). Error bars represent 1 s.d. in parameter estimation of the phase, when polariton intensity is fit to equation (2) (see ‘Methods' section). (**e**) Same as **d** at *B*_*z*_=−6 T, blue: 0.011 *V*_G_+0.0089 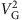, yellow: −0.014 *V*_G_+0.0084 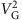.

To demonstrate the validity of the description of photon/polariton dynamics as determined by a gauge potential, we perform an interference experiment where we measure the phase accumulated by polaritons propagating for a length *l* inside the sample relative to a fixed phase reference (see Fig. 3a and ‘Methods' section for detailed information about the interference experiment). An interference image obtained at *E*_ext_=0 is shown in Fig. 3b. Changes in the interference pattern with *y* at two different values of *E*_ext_ are illustrated in Fig. 3c.

A plot of the phase change of polaritons with *E*_ext_ for different directions of excitation at *B*_*z*_=6 T is shown in Fig. 3d. The data show that at a fixed *E*_ext_ and at the same excitation frequency, the phase accumulated for polaritons propagating along 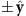 directions differ, and that this phase difference depends on the sign of *E*_ext_ and *B*_*z*_. The kinetic momentum of polaritons with a fixed energy is modified by the absence or presence of a constant vector potential so that the additional accumulated phase for travelling a distance *l* is given by 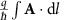. At a fixed *E*_ext_, the difference between the phase accumulated when exciting with 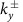 allows us to avoid phase changes due to the Stark effect and to directly obtain 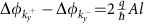. The fact that 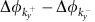 depends both on the sign of the electric field as well as the magnetic field is another manifestation of the validity of the description with *qA*. Since the phase shift as a function of *E*_ext_ can be modelled with a second-order polynomial in *E*_ext_, the dc-Stark effect is the dominant underlying mechanism for the phase shift. Figure 3d shows that polaritons acquire a phase shift of ∼0.25 radians due to *qA* as they propagate an average distance *l*=9 μm.

## Discussion

The parameters of our experiment already ensure significant phase accumulation over the lattice constant (≥2.4 μm) of state-of-the-art polariton lattices^8^ or average separation (∼4 μm) of coupled polariton microcavities^26^. Choosing detunings that yield predominantly exciton-like lower polaritons will further increase *qA*. In addition, employing structures with longer polariton lifetimes^27^ will allow the realization of polariton lattices with larger lattice constants. With these advances, it should be possible to design structures where the accumulated polariton phase due to *qA* in a unit cell could be on the order of *π*.

Together with strong photon–photon interactions, realization of tunable artificial gauge fields is key for ongoing research aimed at the observation of topological order in driven-dissipative photonic systems^28^,^29^. Cavity-polaritons constitute particularly promising candidates in this endeavour since their partly excitonic nature enhances interactions^19^,^20^,^21^, and in an external magnetic field interplay of exciton Zeeman splitting and photonic spin-orbit coupling, can lead to opening of non-trivial energy gaps^30^,^31^. Our work demonstrates that the same polariton system may be complemented by the realization of tunable gauge fields, if an in-plane electric-field gradient is introduced in the QW. While such gradients are manifest in most gated structures, creation of a constant artificial magnetic field over a region of several microns should be possible in specially designed structures. We envision that lattices of dipolaritons^32^ would provide a very promising avenue towards this endeavour: the use of an in-plane magnetic field in this case would effect a gauge potential that is substantially enhanced by the large dipolariton polarizability and strong external electric fields along the growth direction^13^,^14^. The gradients of the latter in turn could be used to generate fluxes approaching magnetic flux quantum in a plaquette of area ∼5 × 5 μm^2^. (Di)Polaritons that are subject to complex electric field distributions enabling large, tunable and inhomogeneous effective gauge field profiles^33^ would constitute a novel feature in the exploration of topologically non-trivial non-equilibrium quantum systems.

## Methods

### Sample

Our sample consists of three layers of 9.6 nm-thick In_0.04_Ga_0.96_As QWs sandwiched between two distributed Bragg reflectors (DBRs) formed by 20 (top) and 22 (bottom) pairs of *λ*/4 thick AlAs/GaAs layers. The GaAs spacer layer containing QWs between the top and bottom DBRs is 1*λ* thick. Spectral linewidths, measured at a position where the cavity mode is far red-detuned from the exciton mode, indicate that the cavity has a *Q* factor exceeding 10^4^ (full-width at half-maximum linewidth 0.11 meV). The exciton emission spectrum at the same position has a full-width at half-maximum linewidth less than 1.2 meV.

Using electron beam lithography and lift-off techniques, we have defined rectangular metal (10 nm Ti, 210 nm Au) pads that are 700 × 120 μm. All of the experiments reported in this paper were carried out between two such pads that are separated by 30 μm. These pads are wire bonded onto a chip carrier, and were driven by a high-voltage amplifier (Falco Systems WMA-300) to apply electric fields to the QW. The position on the sample is chosen such that for 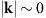 and *B*_*z*_=0 the detuning of the cavity mode energy from the exciton resonance is 0.09 meV, which is small compared to the exciton cavity coupling strength 5.2 meV.

### In-plane electric field

As described in Fig. 1a, we apply an electric potential *V*_G_ between two gates to create *E*_ext_. We find a good agreement between our data and a numerical calculation for the polariton behaviour (see Supplementary Notes 1 and 4) with the electric field *E*_ext_=0.84 *V*_G_/(30 μm). Since the two quantities are linearly related, we use *E*_ext_ and *V*_G_ interchangeably. For all experiments that involve a non-zero in-plane electric field, we acquire data in a pulse sequence (see Supplementary Note 2). This sequence is applied to avoid charge accumulation-induced variation in the actual electric field.

### *k*-resolved photoluminescence spectra

To measure the *k*-resolved photoluminescence (PL) spectra, polaritons are created non-resonantly by a 780 nm continuous-wave diode laser. The incoming collimated laser beam passes through a high numerical aperture (NA) lens (NA=0.68) so that the beam is focused on the sample plane. The PL signals from the sample also pass through the same lens. The back aperture of the high NA lens is the Fourier plane of emission. Emission around a particular in-plane momentum (*k*_*x*_, *k*_*y*_) is collected by coupling the light from a small spot in the Fourier plane into a single-mode fibre which is connected to a spectrometer. To obtain the polariton energy dispersion with respect to the in-plane wavevector *k*_*y*_, we varied the position of the single-mode fibre, which changes the collection spot on the Fourier plane. For each position that corresponds to *k*_*x*_=0 and a *k*_*y*_ value, we record the PL spectrum on the spectrometer. The incident 780 nm laser is blocked using a 800 nm long-pass filter at the entrance to the spectrometer.

### Polariton interference measurement

We use the set-up depicted in Fig. 3a to create two beams that are incident with different in-plane momenta (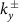 and *k*=0) and are separated by *l*. The laser energy is chosen to be nearly resonant with the polariton modes with 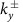. Owing to the steep polariton dispersion, at the same energy, the *k*=0 beam is off resonance from the polariton modes; the photons in this beam are therefore reflected by the top mirror without being subject to *qA*. Photons in the 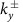 beam are converted into a propagating polariton cloud. As polaritons propagate towards the position of the *k*=0 beam, they accumulate an additional phase due to *qA*. Propagating polaritons continue to emit photons out of the sample, and when these photons overlap with those from the *k*=0 beam they interfere, allowing us to measure changes in the accumulated phase.

We model the light intensity in the region where the interference occurs as the sum of a Gaussian (*k*=0 beam) and a plane wave (polaritons propagating with *k*_*y*_):


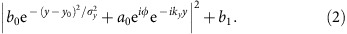


We independently determine *b*_0_, *σ*_*y*_ and *y*_0_ using images obtained at very high applied electric field, for which the polariton term is negligible 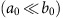, and fix *k*_*y*_. For each *E*_ext_, we fit the detected polariton intensity to equation (2) to find values of *a*_0_, *b*_1_ and *φ*.

Since our excitation beams have a finite size, they have a finite width in *k*_*y*_ (0.45 μm^−1^), therefore, the exact *k*_*y*_ value of the polaritons that are excited is determined by the energy of the excitation beam. For a fixed excitation laser energy, changes in *E*_ext_ will shift the dispersion relation so that 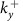
Δ*k*_*y*_ is resonant with the excitation laser. These changes (due to *qA* or Stark effect) in the dispersion relation show up as phase changes in our experiments, Δ*φ*=*l*Δ*k*_*y*_ where *l* is the mean distance from the excitation spot to the interference region. Since we are interested in how the phase changes with *E*_ext_ and the absolute phase between the two beams is not determined, we set the phase at *E*_ext_=0 to 0 radian.

### Data availability

The data that support the plots within this paper and other findings of this study are available from the corresponding author upon reasonable request.

## Additional information

**How to cite this article:** Lim, H.-T. *et al*. Electrically tunable artificial gauge potential for polaritons. *Nat. Commun.* **8,** 14540 doi: 10.1038/ncomms14540 (2017).

**Publisher's note**: Springer Nature remains neutral with regard to jurisdictional claims in published maps and institutional affiliations.

## Supplementary Material

Supplementary Information Supplementary Notes, Supplementary Figures and Supplementary References
