# Supplementary material for: Electrically tunable artificial gauge potential for polaritons
Source: Nat Commun. 2017 Feb 23;8:14540. doi: 10.1038/ncomms14540 (PMC5331219; doi:10.1038/ncomms14540)
Supplement: Supplementary Information — Supplementary Notes, Supplementary Figures and Supplementary References [file ncomms14540-s1.pdf]

Supplementary Note 1. SIMULATION OF ELECTRIC FIELD DISTRIBUTION

To confirm that the metal pad configuration described in the Methods section of the main text provides a uniform and controllable electric field distribution we performed a 2D electrostatic simulation of the dielectric environment in COMSOL. We modelled the AlAs/GaAs/InGaAs environment as a single dielectric with $\epsilon = 12.9$. The gates are modelled as 200 nm thick metals with a 30 µm gap, and the electric potential is constant and fixed on their boundary. The electric potential, and the electric field distribution when one of the gates is applied 1 V and the other is kept at 0 V is illustrated in Supplementary Figure 1.

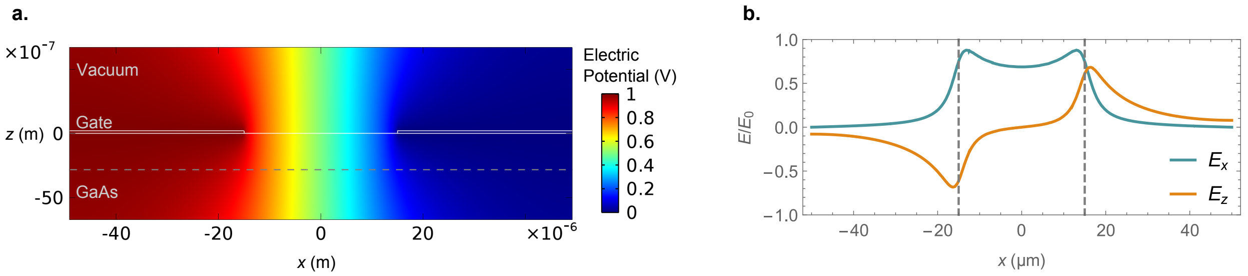

Supplementary Figure 1: **Simulation of the electric field profile. a.** Calculated electric potential for 1 V applied to the left gate while the right gate is kept at 0 V. White solid lines show the boundaries of different regions containing different materials that are modelled (labelled on figure). Dashed grey line indicates the position of the electric field line cuts. **b.** The line cut in **a** showing the electric field components at $z = -2.7\,\mu\text{m}$ which corresponds to the position of the quantum well. $E_0 = 1/30$ V µm^{-1}. Vertical grey dashed lines indicate the position of the edges of the gates.

In the simulations we find that the electric field within a few microns from the centre of the gap of the gates at a depth that corresponds to the quantum well (QW) position should be relatively uniform and along the x direction. In this uniform region the electric field is 0.69 $V_G/(30\,\mu\text{m})$ where V_G is the applied voltage between the gates.

Supplementary Note 2. EXPERIMENTAL DETAILS

We find that in measurements where an electric field is applied, the results are affected by the duration, intensity, and energy of excitation lasers. We believe these changes are due to excess charge carriers that are optically created that screen the externally applied electric field. In order to avoid such effects, in experiments where an applied electric field is needed, we perform experiments with a weak laser (New Focus TLB-6716, < 100 pW for reflection experiments and ≤ 40 nW for

interference experiments) and, to reduce light exposure further, we apply a sequence of low duty cycle laser pulses. These laser pulses are timed with a voltage pulse sequence that alternates the sign of the applied voltage between the gates to ensure that the average electric field applied to the sample remains 0.

For reflection and interference experiments we use the pulse sequence illustrated in Supplementary Figure 2. To acquire reflection or interference data corresponding to a target voltage V_{target} we ensure that the counter or the camera (PointGray Grasshopper 3 41C6NIR) is gated so that data from the time interval when the applied voltage is V_{target} is recorded. Voltage pulse lengths are chosen to be 22.4 µs. To limit the exposure time of the laser on the sample, the excitation laser is modulated using an acousto-optic modulator (AOM, double pass, extinction $\geq$ 50 dB). The excitation laser is switched on by the AOM only around the time interval where V_{target} is applied. Following these voltage pulses we apply 20 pairs of the high voltage pulse $\pm V_{\mathrm{high}} = \pm 10$ V to remove the remaining charge carriers created during the measurement so that these excessive charge carriers do not affect the following measurement. Our data is not sensitive to the exact values used in the sequence, for example voltage value used for V_{high} pulses, the number of pairs of V_{high} pulses used, voltage pulse lengths, and laser illumination duration. Hence these values can be varied without affecting the results, but some form of this pulse sequence is necessary to obtain consistent electric field-dependent data. For all the electric field-dependent data reported in this paper, we use the sequence described in this paragraph.

Each data point measured presented in the main text is an average of data recorded in multiple runs of the pulse sequence. For example the reflection data shown in Figure 1 (main text) consists of 4,500 repetitions of the pulse sequence for each target voltage value, whereas the interference image shown in Figure 3 (main text) is formed by acquiring and summing 107,000 frames from the camera; each frame corresponds to a repetition of the pulse sequence. Data acquisition from the camera is carried out in blocks of 1,000 frames where pixel values from all 1,000 frames are added to form a single image. Following a block of data acquisition we do an equal acquisition with 1,000 frames with the illumination laser turned off and record the difference between the two images to remove any noise due to a persistent background in the images.

Polarization of the excitation beams is fixed to be linear (close to p-polarization) for all experiments. In the reflection measurement we measure the reflected light intensity without any additional polarization filtering. In polariton interference experiment the polarizer in front of the camera transmits the light nearly orthogonally polarized to the incident beams to observe high visibility interference images. This nearly orthogonal polarization configuration significantly at-

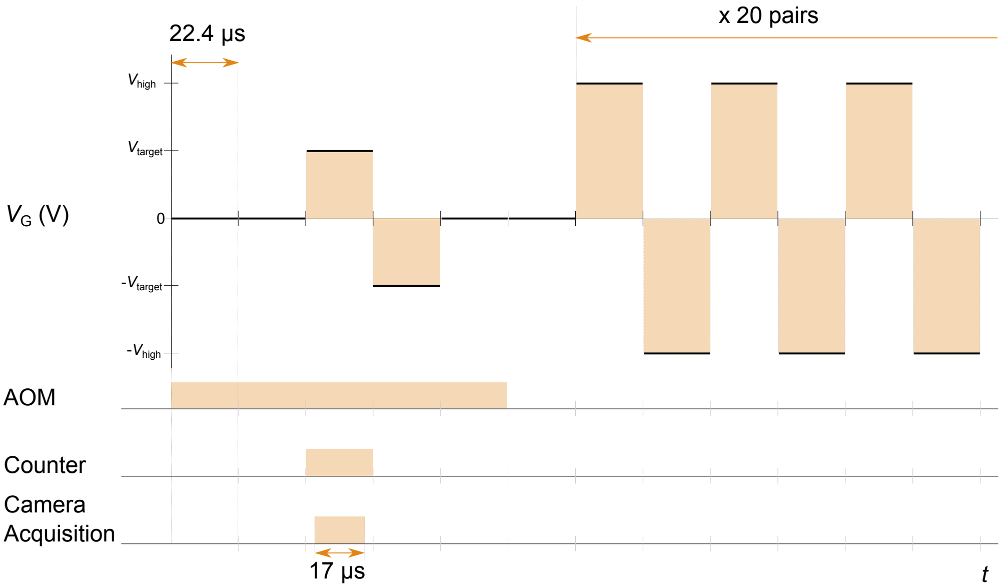

Supplementary Figure 2: **Pulse sequence.** We acquire the data only when the target voltage V_{target} is applied. This is ensured by timing the exposure of the camera, and gating the counter. Each voltage pulse is 22.4 µs. The laser is incident on the sample only when V_{G} is $\pm V_{\mathrm{target}}$ or 0.

tenuates the intensity of the reflected excitation lasers, however it does not significantly attenuate the elliptically polarized emission from the polariton transitions at high magnetic fields.

Supplementary Note 3. EXTRACTION OF POLARITON AND EXPERIMENTAL PARAMETERS

Magnetic field dependent k resolved photoluminescence (PL) spectra measurements allow measurement of many parameters that allow us to accurately model the polariton behaviour at high magnetic fields [1].

Experimental results for the PL dispersion measurements at $B_z = 0$ T, 3 T, and 6 T are shown in Supplementary Figure 3. For each k_y value we fit two Lorentzian lineshapes to determine the lower polariton (LP, lower energy eigenstate) and upper polariton (UP, higher energy eigenstate) energies. We then fit the exciton-polariton energy dispersion with respect to the in-plane momentum $\hbar k_y$ to:

$$\epsilon_{\mathrm{LP,UP}}\left(k_y\right) = \frac{1}{2}\left[\epsilon_{\mathrm{cav}}\left(k_y\right) + \epsilon_{\mathrm{exc}}\left(k_y\right)\right] \pm \frac{1}{2}\sqrt{\left[\epsilon_{\mathrm{cav}}\left(k_y\right) - \epsilon_{\mathrm{exc}}\left(k_y\right)\right]^2 + \Omega^2}, \tag{1}$$

where Ω is the exciton cavity coupling strength that we refer to as Rabi energy, and we assume that $\epsilon_{\mathrm{exc}}\left(k_y\right) \simeq \epsilon_{\mathrm{exc}}(0)$ and $\epsilon_{\mathrm{cav}}\left(k_y\right) \simeq \epsilon_{\mathrm{cav}}(0) + \frac{1}{2m_{\mathrm{cav}}}\left(\hbar k_y\right)^2$. Based on this relation, we can extract

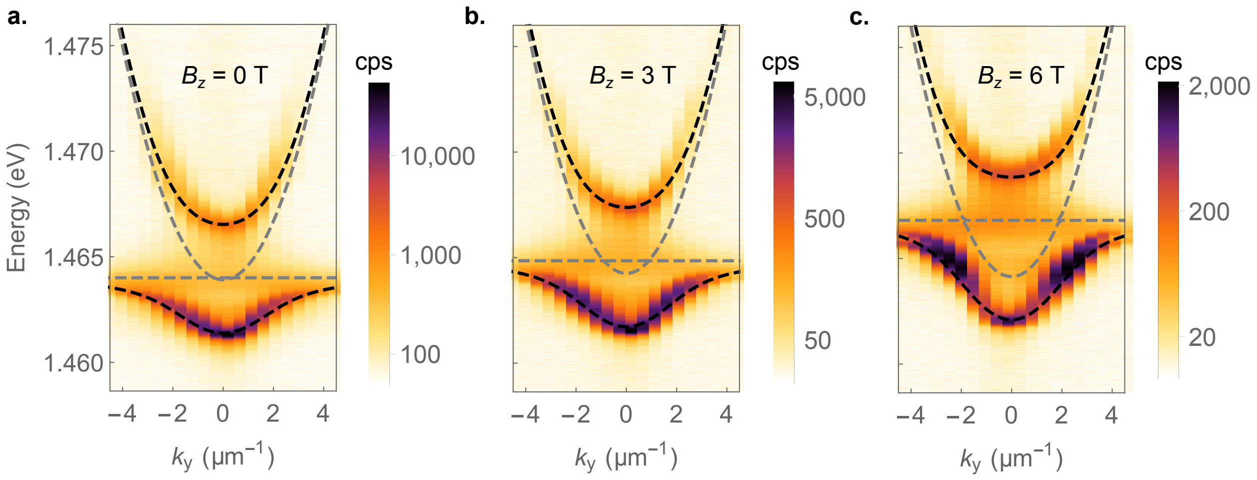

Supplementary Figure 3: k_y **resolved photoluminescence spectra of exciton-polaritons. a.** Spectra for $B_z = 0$ T. **b.** Spectra for $B_z = 3$ T. **c.** Spectra for $B_z = 6$ T. cps stands for counts per second. The grey dashed lines are the bare cavity (curved) and exciton (flat) energy dispersion and the black dashed lines correspond to the upper and lower polariton resonances. As B_z increases from 0 T to 6 T, the cavity energy remains the same while the exciton energy increases due to the diamagnetic shift. Two peaks are present for lower polariton energy dispersion at high magnetic fields (panel **c**) and large wavevectors, which we attribute to the influence of Zeeman splitting of the exciton transitions.

$\epsilon_{\text{exc}}(0)$, $\epsilon_{\text{cav}}(0)$, and Ω from the experimental result at each magnetic field, these extracted values are shown in Supplementary Figure 4. We will use these values in Supplementary Note 4 when describing the detailed model we use to describe polariton behaviour.

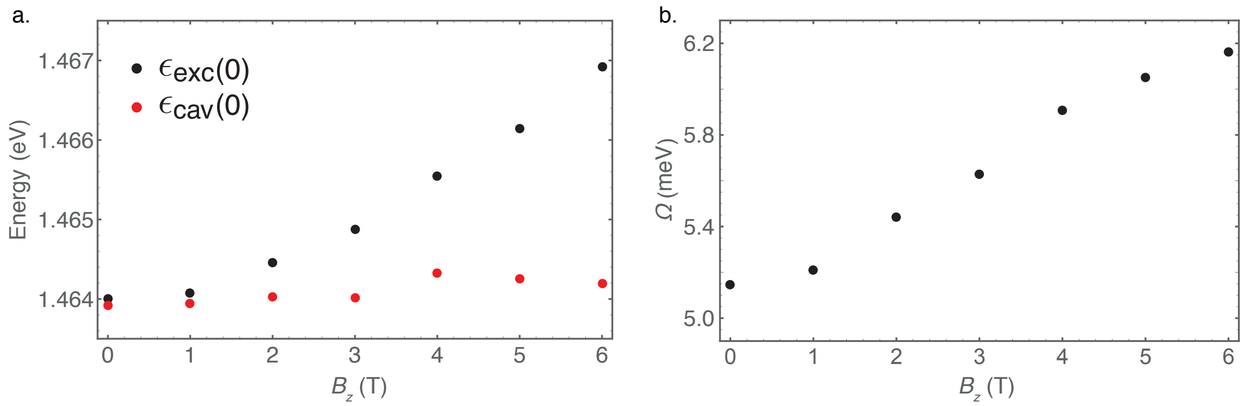

Supplementary Figure 4: **Magnetic field dependence of exciton energy, cavity energy and Rabi energy. a.** The exciton energy ϵ_{exc} (black) increases with B_z while the cavity mode energy ϵ_{cav} (red) is more or less constant regardless of B_z. **b.** Rabi energy Ω increases with B_z.

k_y distribution of detection and excitation beams

Due to the finite size of the beams used, a k_y uncertainty, which we quantify below, is present for both excitation and detection beams. With this uncertainty, we expect emission spectra measured around a mean k_y value to be asymmetric, and the linewidth of the spectra to depend on this mean value. In particular our collection fibre acts as a filter and selects a distribution of k_y values. We model the field distribution as a probability density function given by $f_{k_y}(k'_y) = \frac{1}{\sqrt{2\sigma_k^2\pi}} e^{-\frac{(k'_y - k_y)^2}{2\sigma_k}}$. We assume a polariton resonance at $\epsilon_{\mathrm{LP}}(k'_y)$ leads to an emission intensity lineshape given by $A\frac{\Gamma/2}{(\epsilon - \epsilon_{\mathrm{LP}}(k'_y))^2 + (\Gamma/2)^2}$ where A is a constant characterizing intensity of polariton emission. The measured spectrum is then modelled by

$$I(\epsilon, k_y) = \int dk'_y |f_{k_y}(k'_y)|^2 \left[A \frac{\Gamma/2}{(\epsilon - \epsilon_{\mathrm{LP}}(k'_y))^2 + (\Gamma/2)^2} \right] \quad (2)$$

With $\sigma_k = 0.5 \times 10^6$ m^{-1}, and the linewidth $\Gamma = 120\,\mu\mathrm{eV}$ we obtain a reasonable agreement between the model and the observed lineshapes. Supplementary Figure 5 illustrates the resulting asymmetric lineshapes as well as the change in the expected linewidths with k_y. Note that at high k_y values there is a high energy tail in the emission lineshapes that is not captured by this model. This tail occurs at energies close to the bare exciton resonance.

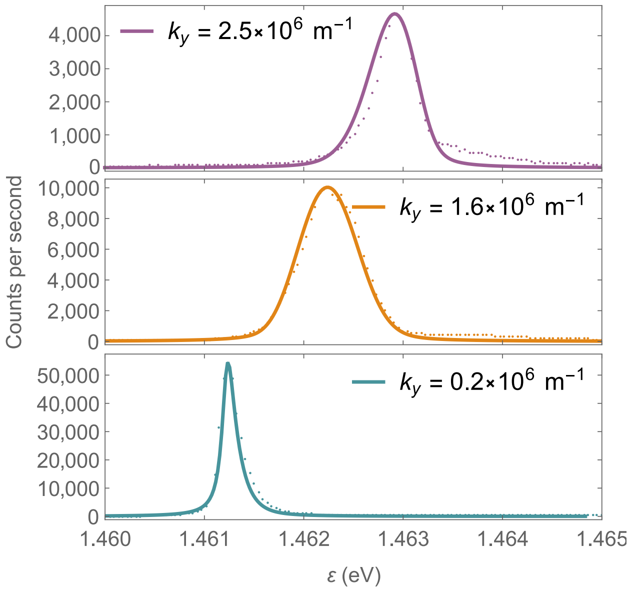

Supplementary Figure 5: **Changes in PL emission lineshape with k_y value.** Dots are horizontal line cuts of the data shown in Supplementary Figure 3 at $B_z = 0$ T, solid lines are estimated lineshapes using Supplementary Equation 2.

To measure the k_y distribution of the excitation beams used for the interference experiment (main text Figure 3) we slightly modify the interference experiment performed. We detune the excitation laser to the red of the polariton resonances so that the laser does not excite any polaritons, and we change the polarizer angle in front of the camera such that two beams have equal detected intensities. We also change the distance between the high NA lens and the sample so that the two beams overlap at the sample surface. With the sample illuminated by the k_y^- beam and the $k = 0$ beam, an image of the light intensity at the surface shows an interference pattern illustrated in Supplementary Figure 6. We emphasize again that, unlike the experiments in the main text, here polaritons have not been excited, and the image purely shows the interference of two laser beams. This interference pattern is due to the spatially varying phase between two beams, for simplicity (considering only y direction) we model this interference as the interference of two beams that are defined by their field distributions $f_{k_y^-}(k_y')$ and $f_0(k_y')$. To extract k_y^- and σ_k^- we take a 2D Fourier Transform of the intensity image, and analyze the line cut at $k_x = 0$. From the position of the two Gaussian peaks we find $k_y^- = -2.9 \times 10^6$ m^{-1}. From their widths we find $\sigma_k^- = 0.4 \times 10^6$ m^{-1}. Acquiring and analyzing a dataset using k_y^+ excitation beam we find $k_y^+ = 2.7 \times 10^6$ m^{-1} and $\sigma_k^+ = 0.5 \times 10^6$ m^{-1}.

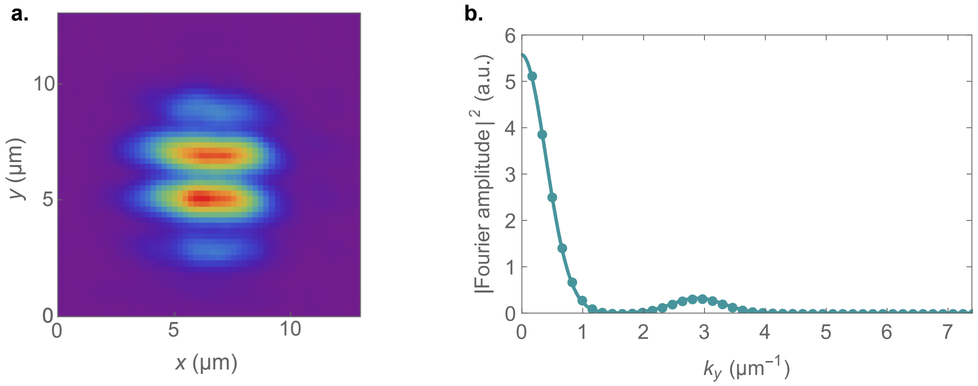

Supplementary Figure 6: **Estimation of k_y from interference images. a.** Interference images between the $k = 0$ beam and the k_y^- beam. **b.** Line cut of the 2D Fourier Transform of the image in a, showing the positive k_y values at $k_x = 0$. Solid lines are obtained by fitting a sum of two Gaussian functions one centred at $k_y = 0$ the other centred at $k_y = k_y^-$.

Extracting electric field dependent energy shift of polaritons

Due to the fibre coupling we employ, the excitation laser intensity exhibits significant changes (up to 25 %) as its energy is tuned. Since the excitation intensity is less than 100 pW, we do not

expect any changes in the polariton behaviour due to these intensity variations. To be able to fit, and visualize the underlying changes in intensity due to the excitation of polariton resonances, we calculate and plot the ratio of the recorded intensity to the average of the intensity of the data obtained for the six highest voltage values applied for each laser energy. For example in Figure 1d in the main text each horizontal line is obtained by dividing the reflected intensity for each voltage by the average intensity of the reflection at 11.04, 11.52, 12, 12.48, 12.96 and 13.44 V.

This normalization procedure however affects the lineshape of the reflection data. The procedure above (due to the Stark shift) effectively calculates the ratio by dividing the average of data points of reflected intensity at high energy tails of the polariton resonance. If these points overlap with the tail due to the asymmetric lineshape (observed in the PL spectra, Supplementary Figure 5) that extends to high energies, or overlaps with the polariton resonance, the reflection ratio at low energies can have values higher than 1. To account for this lineshape that is asymmetric in energy relative to the polariton resonance, we include a smooth step function in our fits. The fitting function we use at low magnetic field is given by :

$$R_{\text{low}}(\epsilon) = b_r + a_r \frac{(\mathit{\Gamma}/2)^2}{(\epsilon - \epsilon_0)^2 + (\mathit{\Gamma}/2)^2} + a_{\text{step}} \tanh\left(\frac{\epsilon - \epsilon_0}{\epsilon_{\text{step}}}\right). \tag{3}$$

We use for our fits $\epsilon_{\text{step}} = 0.2$ meV. At high magnetic fields the data exhibits two closely spaced dips that we attribute to the interplay of the Zeeman splitting of the exciton transitions and the TE-TM splitting of the photonic modes, and use:

$$\begin{aligned} R_{\text{high}}(\epsilon) = b_r \ &+\ a_{r,+} \frac{(\mathit{\Gamma}_+/2)^2}{(\epsilon - \epsilon_0 + \Delta)^2 + (\mathit{\Gamma}_+/2)^2} + a_{r,-} \frac{(\mathit{\Gamma}_-/2)^2}{(\epsilon - \epsilon_0 - \Delta)^2 + (\mathit{\Gamma}_-/2)^2} \\ &+\ a_{\text{step}} \tanh\left(\frac{\epsilon - \epsilon_0}{\epsilon_{\text{step}}}\right). \end{aligned} \tag{4}$$

We expect, due to the finite TE-TM splitting at the $k_y^{\pm}$ values that we perform the measurements that the transitions will be elliptically polarized and the two dips might be of different amplitude.

Supplementary Note 4. MODEL OF POLARITONS UNDER MAGNETIC AND ELECTRIC FIELDS

We model the behaviour of polaritons in our system by numerically determining the eigenvalues and the associated wavefunctions of a Schrödinger equation for the excitons in the system. We compare the measured observables obtained in various experiments with the results of calculations based on the numerical study both to extract parameters that describe the exciton system, as well as to verify the physical origin of the change in the observables.

The Hamiltonian for the 2D exciton relative motion wave function $\psi(\mathbf{r})$ is given by [2, 3]

$$H = -\frac{\hbar^2}{2\mu}\Delta_r + \frac{eB_z}{2\eta}\hat{L}_z + \frac{P^2}{2M} + \frac{1}{2\mu}\left(\frac{B_z}{2}\right)^2 r^2 + \frac{e}{M}\mathbf{P}\times(B_z\hat{z})\cdot\mathbf{r} + V(r) + e(E_{\text{ext}}\hat{x})\cdot\mathbf{r}. \quad (5)$$

where the Hamiltonian is described in SI unit and $\mathbf{r} = \mathbf{r}_\text{e} - \mathbf{r}_\text{h}$ is the relative coordinate, $\mathbf{R} = (m_\text{e}\mathbf{r}_\text{e} + m_\text{h}\mathbf{r}_\text{h}) / (m_\text{e} + m_\text{h})$ is the centre of mass (CM) coordinate, $\hat{L}_z = i\hbar\hat{z}\times\nabla_r$ is the angular momentum operator in the z (growth) direction, $\mathbf{P}$ is the exciton magnetic CM momentum, which is an eigenvalue of the magnetic momentum operator $\hat{P} = -i\hbar\nabla_R - e\mathbf{B}\times\mathbf{r}/2$. Note that $\mathbf{P}$ is the conserved momentum of the exciton in a magnetic field and associated with translational invariance in the plane of the quantum well and is identical to the CM momentum at $B = 0$ [2]. Hence the exciton excited by a photon has $\mathbf{P} = \hbar\mathbf{k}$, which is the same as the in-plane momentum of the photon. $E_\text{ext}\hat{x}$ is the applied electric field, B_z the magnetic field in the growth direction, $\mu^{-1} = m_\text{e}^{-1} + m_\text{h}^{-1}$, $\eta = m_\text{e}^{-1} - m_\text{h}^{-1}$, and $M = m_\text{e} + m_\text{h}$ is the electron-hole pair total mass (total exciton mass).

We ignore the $\frac{eB}{2\eta}\hat{L}_z$ term in Supplementary Equation 5 since we are dealing with the ground state of the 2D exciton wave function, which is spatially symmetric ($1s$ state for $B_z = 0$). We also ignore the centre of mass kinetic energy term $(\hbar\mathbf{k})^2/(2M)$ since we fix the magnetic CM momentum $\hbar\mathbf{k}$ in the experiment. Thus, this term gives a constant energy shift.

The effective Coulomb potential for a QW with finite width d is [4–6]

$$V(r) = -\frac{e^2}{4\pi\varepsilon\varepsilon_0}\int_0^d\int_0^d dz_\text{e}dz_\text{h}\frac{|U_\text{e}(z_\text{e})|^2\,|U_\text{h}(z_\text{h})|^2}{\sqrt{r^2 + (z_\text{e} - z_\text{h})^2}}, \quad (6)$$

where ε is the relative permittivity of the material, ε_0 is the vacuum permittivity, and we assume that the QW has infinite barriers. The wavefuntions of the electron and hole in the growth direction is given by $U_i(z_i) = \sqrt{\frac{2}{d}}\sin\left(\frac{z_i}{d}\pi\right)$ where i is either e or h. If we introduce the variable $u = (z_\text{e} - z_\text{h})/d$ and $v = (z_\text{e} + z_\text{h})/d$, then Supplementary Equation 6 can be written as:

$$\begin{aligned} V(r) &= -\frac{1}{\pi\varepsilon\varepsilon_0 d}\int_0^1\int_u^{2-u} dv du\frac{\sin^2\left(\frac{u+v}{2}\pi\right)\sin^2\left(\frac{u-v}{2}\pi\right)}{\sqrt{\left(\frac{r}{d}\right)^2 + u^2}} \\ &= -\frac{1}{4\pi\varepsilon\varepsilon_0 d}\int_0^1 du\frac{(1-u)\left[2+\cos(2\pi u)\right] + \frac{3}{2\pi}\sin(2\pi u)}{\sqrt{\left(\frac{r}{d}\right)^2 + u^2}} \\ &\equiv -\frac{1}{4\pi\varepsilon\varepsilon_0}h(r). \end{aligned} \quad (7)$$

We use Mathematica's finite element method for numerically solving the Schrödinger equation with the Hamiltonian given by Supplementary Equation 5 to obtain the energy and the wave function of the lowest energy state. The computation region is limited to $r_\text{max} = 10\ a_0$ $(0 \le |r| \le$

r_{max}, and $a_0 = 4\pi\varepsilon\varepsilon_0\hbar^2/\mu e^2$, 3D Bohr radius of the exciton), which is large enough that the lowest eigenvalue of the equation does not change (up to six digits) as r_{max} increases.

The physical parameter values used in this calculation are summarized in Supplementary Table I. m_0 is the electron mass and we choose the remaining parameters such as k_y, μ, and M, as we discuss below, to best match with our experimental data.

Parameter	Symbol	Value
Relative permittivity (GaAs)	ε	12.9
Quantum well width	d	9.6 nm
In-plane wavevector	$k_y^{\pm}$	$\pm 2.8 \times 10^6$ m^{-1}
Exciton effective (reduced) mass	μ	0.04 m_0
Total exciton mass	M	0.07 m_0
Cavity effective mass	m_{cav}	6×10^{-5} m_0

Supplementary Table I: **Physical parameter values.** m_0 is the electron mass.

Diamagnetic shift of excitons

We calculate the energy of the lowest energy eigenstate of Supplementary Equation 5 with $E_{\text{ext}} = 0$ as a function of magnetic field, B_z, and compare the shift of energy of this state with the shift of the extracted exciton transition energy obtained in Supplementary Note 3. To match with the experimental results on the exciton energy change we vary the reduced mass of the exciton μ. See Supplementary Figure 7 for the comparison between the theory with various μ and the experimental results. Based on the results on Supplementary Figure 7, we choose $\mu \sim 0.04\ m_0$. Then, the binding energy of the exciton with no external magnetic and electric field is 7.94 meV.

Effect of the applied electric field on polaritons

An electric field alters the exciton energy thereby changing the polariton resonance energy. In addition polarization of the exciton due to E_{ext} reduces the electron hole overlap, leading to a reduced Ω, which also changes the polariton resonance energy. To model the polariton behaviour we use Supplementary Equation 1 where Ω and ϵ_{exc} depend on both B_z and E_{ext}. Shifts in $\epsilon_{\text{exc}}\left(B_z, E_{\text{ext}}\right)$ are found by identifying the energy shifts of the lowest energy eigenstate of Supplementary Equation (5). The cavity photon energy is given as $\epsilon_{\text{cav}}\left(k_y\right) \simeq \epsilon_{\text{cav}}\left(0\right) + \frac{\hbar^2}{2m_{\text{cav}}}k_y^2$ where

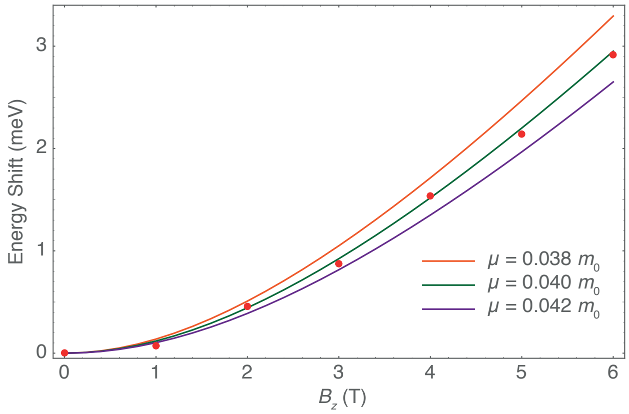

Supplementary Figure 7: **Exciton diamagnetic shift.** As the magnetic field increases, the exciton energy increases. Red data points are the experimental results and the red, green, and purple lines are obtained from the numerical solution to Supplementary Equation 5 with $\mu = 0.038\,m_0,\ 0.040\,m_0,\ 0.042\,m_0$, respectively.

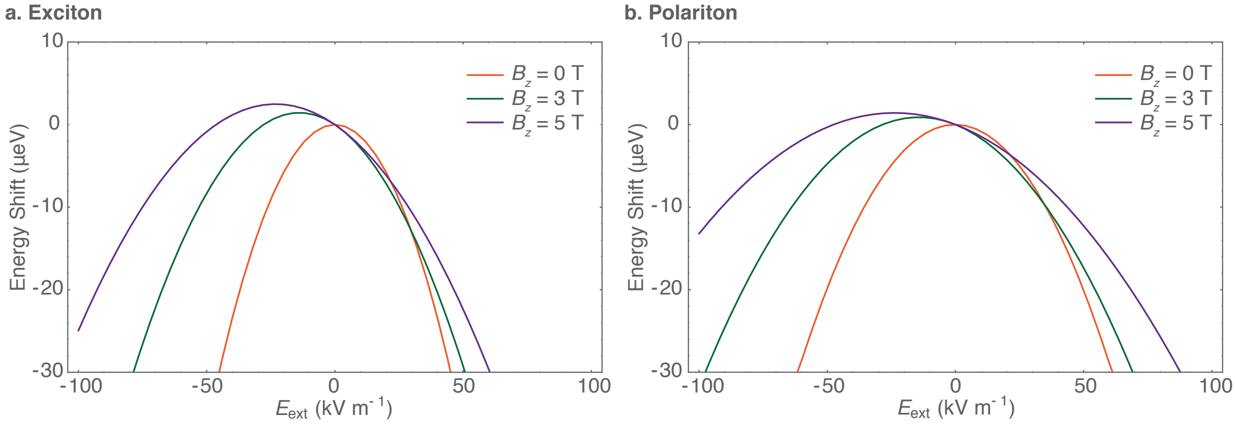

Supplementary Figure 8: **Energy shift with applied electric field. a.** Exciton and **b.** Lower polariton energy shows parabolic shifts against the applied electric field E_{ext}. The maximum exciton energy is obtained at $E_{\mathrm{ext}} = \tilde{E}_{\mathrm{ext}}$. The polarizability of the exciton, also, decreases as B_z increases. The energy of the exciton changes more rapidly than the energy of the polariton. The red, green, and purple lines correspond to $B =$ 0 T, 3 T, and 5 T, respectively. $k_y = 2.8 \times 10^6$ m^{-1} and $M = 0.07\ m_0$.

we assume E_{ext} or B_z does not change the cavity resonance and dispersion. Changes in Ω are calculated as indicated in the previous section. Supplementary Figure 8b shows the lower polariton energy shift with E_{ext} at B_z = 0 T, 3 T, and 5 T.

We fit the polariton energy shift with E_{ext} to a second order polynomial of E_{ext}, i.e. $\epsilon_{\mathrm{LP}}(E_{\mathrm{ext}}) \simeq \epsilon_{\mathrm{LP}}(0) - dE_{\mathrm{ext}} - \alpha E_{\mathrm{ext}}^2$. The comparison between the theoretical result (both of the exciton and polariton cases) and the experiment is shown in Supplementary Figure 10. Increase in $\epsilon_{\mathrm{LP}}(0)$ with B_z is less compared to the calculated increase for the exciton (Supplementary Figure 10a), as the

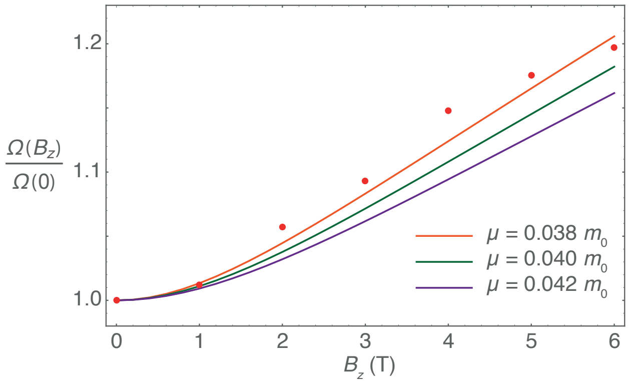

Supplementary Figure 9: **Change in Rabi energy with magnetic field.** The Rabi energy $\Omega\,(B_z, E_{\rm ext})$ is proportional to the norm of the exciton wave function $|\psi\,(r=0)|$ [6], thus, to quantify the changes in Ω we use $\Omega\,(B_z, E_{\rm ext}) = \Omega\,(B_z=0, E_{\rm ext}=0)\,\frac{|\psi(r=0,B_z,E_{\rm ext})|}{|\psi(r=0,B_z=0,E_{\rm ext}=0)|}$. Here we plot the relative Rabi energy changes with B_z. As the magnetic field increases, the Rabi energy increases. Red data points are the experimental results and the red, green, and purple lines the numerical solution to Supplementary Equation 5 with $\mu = 0.038\,m_0,\ 0.040\,m_0,\ 0.042\,m_0$, respectively.

increase in Ω leads to red-shift of the LP energy.

Dependence of the polarizability α on B_z is depicted in Supplementary Figure 10b. We compare the theoretical result of α for polaritons with the experimental results by varying the reduction factor γ of the applied electric field $E_{\rm ext} = \gamma\frac{V_{\rm G}}{30\ \mu{\rm m}}$ where $V_{\rm G}$ is the potential applied to the gate. We find a good agreement with the experimental results when $\gamma = 0.84$. Note that the COMSOL simulation gives $\gamma \sim 0.69$ as discussed in Section I. As shown in Supplementary Figure 10b, both polariton and exciton polarizabilities decrease with B_z but the polarizability of the polariton is smaller than that of the exciton.

Here, as in the main text, we calculate the difference of the effective electric fields $\Delta E_{\rm eff}$ between the case of $k_y^{\pm} = \pm 2.8\times 10^6\ {\rm m}^{-1}$, this is shown as Supplementary Figure 10c. Unlike Supplementary Figures 10a and b, $\Delta E_{\rm eff}$ for polariton and the exciton cases are very similar to each other and the small discrepancy is due to the fact that the Rabi energy is reduced by $E_{\rm ext}$. The electric field difference $\Delta E_{\rm eff}$ shows linear dependence on B_z. Note that we find that the total exciton mass $M \sim 0.07\ m_0$ gives a good agreement between the theoretical and the experimental results. See Supplementary Figure 11 for the simulation results with three different M values.

The difference of the induced dipole moments Δd between $k_y^{\pm}$ with B_z is shown in Supplementary Figure 10d. d corresponds to the induced dipole moment of the exciton or polariton due to the in-plane momentum and B_z. Supplementary Figure 10d shows that the dipole moment of the polariton increases with B_z up to around 3 - 4 T and then decreases with B_z for higher magnetic

fields. A remarkable feature is that the magnetic field at which the dipole moment reaches the maximum value is different for the exciton and the polariton cases, in our sample, the polariton has the maximum value around 3 - 4 T while the exciton has its maximum value around 4 - 5 T. This is due to the fact that the exciton content of the lower polariton decreases with B_z.

Effective vector potential for polaritons

For polaritons at arbitrary detuning, it is not generally possible to write the lower (or upper) polariton dispersion as a free particle with single effective mass. However for a small range of

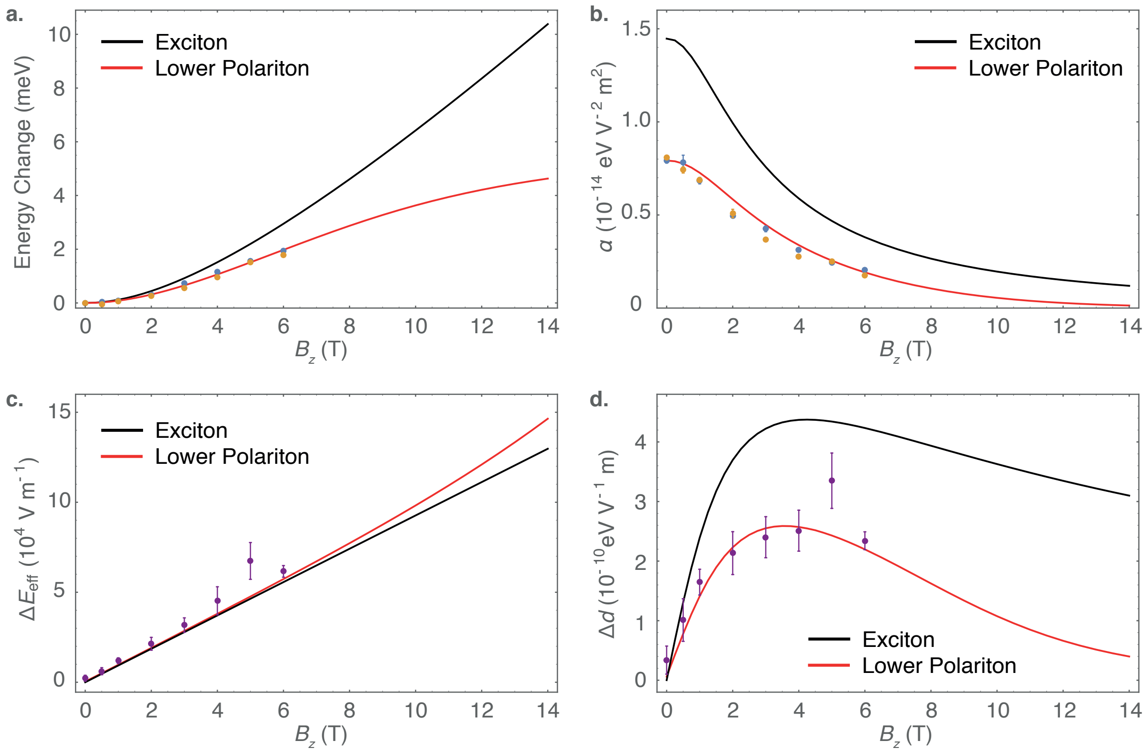

Supplementary Figure 10: **Magnetic field dependence.** For each figure, the black and red lines correspond to the exciton and the polariton simulation results, respectively. The yellow and blue data points corresponds to the experimental data for the polaritons excited with $k_y^+ = 2.7 \times 10^6$ m^{-1} and $k_y^- = -2.9 \times 10^6$ m^{-1}, respectively. **a.** Exciton and polariton energy at $E_{\text{ext}} = 0$ increases with B_z. **b.** The polarizability α decreases with B_z. **c.** Difference between the effective electric fields ΔE_{eff} for k_y^+ and k_y^-. **d.** Difference between the induced dipole moments Δd for k_y^+ and k_y^-. Solid lines are obtained from the numerical solution to Supplementary Equation 5, with $\left|k_y^\pm\right| = 2.8 \times 10^6$ m^{-1} and $M = 0.07\, m_0$. m_0 is the electron mass. Error bars are the estimated standard deviations of the mean for three repetitions of the experiment. Some errorbars are smaller than the marker.

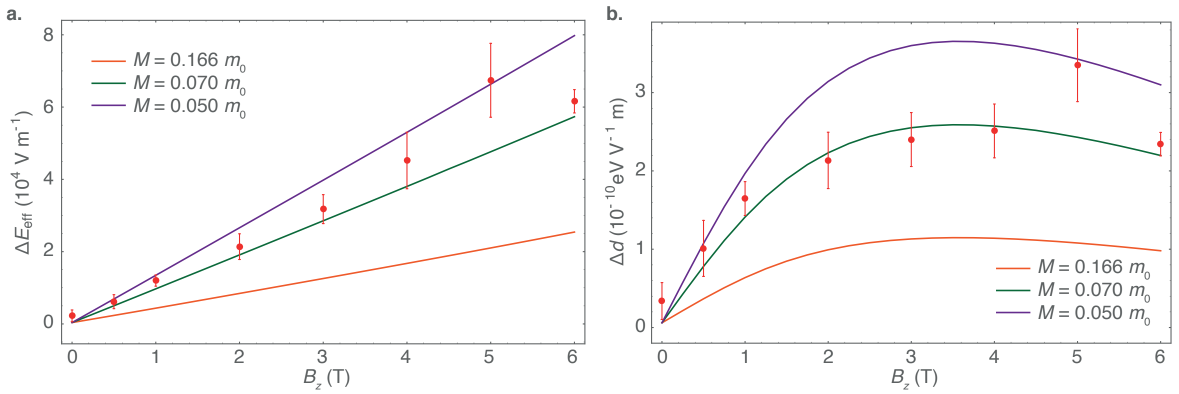

Supplementary Figure 11: **Exciton total mass dependence.** The orange, green and purple lines are obtained from the numerical solution to Supplementary Equation 5 with the exciton mass $M = 0.166\ m_0$, $0.07\ m_0$, and $0.05\ m_0$, respectively. **a.** Difference between the effective electric fields $\Delta E_{\rm eff}$ for k_y^+ and k_y^-. **b.** Difference between the induced dipole moments Δd for k_y^+ and k_y^-. As M increases, both of $\Delta E_{\rm eff}$ and Δd increases. M= $0.07\ m_0$ fits well with our experimental data. Error bars are the estimated standard deviations of the mean for three repetitions of the experiment

wavevector values (δk_y) around a particular wavevector k'_y ($k_y = k'_y + \delta k$) we will show that it is possible to describe polaritons as particles moving in a vector potential. Using the description in the main text, the energy difference between the cavity mode and the exciton mode is:

$$\Delta(k_y) = \epsilon_{\rm cav}(k_y) - \epsilon_{\rm exc}(k_y) = \epsilon_{\rm cav}(0) - \epsilon'_{\rm exc}(0) + \frac{1}{2m_{\rm cav}}(\hbar k_y)^2 - \frac{1}{2M'}(\hbar k_y - qA_{\rm eff})^2 \quad (8)$$

The energy eigenvalues in Supplementary Equation 1 can be re-written as:

$$\epsilon_{\rm LP,UP}(k_y) = \frac{1}{2}\left(\Delta(k_y) \pm \sqrt{\Delta^2(k_y) + \Omega^2}\right) + \epsilon'_{\rm exc}(0) + \frac{1}{2M'}(\hbar k_y - qA_{\rm eff})^2 \quad (9)$$

Assuming $k'_y \gg \delta k_y$ we do a linear expansion in δk_y of $\epsilon_{\rm LP}(k_y)$ and find:

$$\epsilon_{\rm LP}(k_y) \simeq \epsilon(k'_y) + \frac{1}{2m}(\hbar k_y - qA)^2 \quad (10)$$

where $A = \frac{m}{M}2\alpha B_z E_x |X_k|^2$ and $\epsilon(k'_y) = \epsilon_{\rm exc}(k'_y) + \frac{1}{2}\left(\Delta(k'_y) - \sqrt{\Delta^2(k'_y) + \Omega^2}\right) - \frac{1}{2m}\left(\hbar k'_y - qA\right)^2$, $m^{-1} = |C_k|^2 \frac{1}{m_{\rm cav}} + |X_k|^2 \frac{1}{M'}$ with $|C_k|^2 = \frac{1}{2}\left(1 - \frac{\Delta(k'_y)}{\sqrt{\Delta^2(k'_y)+\Omega^2}}\right)$ and $|X_k|^2 = \frac{1}{2}\left(1 + \frac{\Delta(k'_y)}{\sqrt{\Delta^2(k'_y)+\Omega^2}}\right)$ the cavity and exciton Hopfield coefficients.

Supplementary References

[1] Pietka, B. *et al.* Magnetic field tuning of exciton-polaritons in a semiconductor microcavity. *Phys. Rev. B* **91**, 075309 (2015).

[2] Gor'kov, P. & Dzyaloshinskii, I. E. Contribution to the Theory of the Mott Exciton in a Strong Magnetic Field. *Sov. Phys. JETP* **26**, 449–451 (1968).

[3] Lozovik, Y. E., Ovchinnikov, I. V., Volkov, S. Y., Butov, L. V. & Chemla, D. S. Quasi-two-dimensional excitons in finite magnetic fields. *Phys. Rev. B* **65**, 235304 (2002).

[4] Kavokin, A. V. *et al.* The effect of a "Coulomb well" on the absorption and magnetoabsorption spectra of strained InGaAs/GaAs heterostructures. *Semicond.* **31**, 950–960 (1997).

[5] Oliveira, C., Freire, J., Freire, V. & Farias, G. Inhomogeneous broadening arising from interface fluctuations in strained In_xGaAs_{1-x}/GaAs and $(In_uGaAs_{1-u})_v(InP)_{1-v}$/InP quantum wells. *Appl. Surf. Sci.* **234**, 38–44 (2004).

[6] Stepnicki, P., Pietka, B., Morier-Genoud, F., Deveaud, B. & Matuszewski, M. Analytical method for determining quantum well exciton properties in a magnetic field. *Phys. Rev. B* **91**, 195302 (2015).
